# Gut microbiota Modulated by Probiotics and *Garcinia cambogia* Extract Correlate with Weight Gain and Adipocyte Sizes in High Fat-Fed Mice

**DOI:** 10.1038/srep33566

**Published:** 2016-09-23

**Authors:** Jaeyoung Heo, Minseok Seo, Hwanhee Park, Woon Kyu Lee, Le Luo Guan, Joon Yoon, Kelsey Caetano-Anolles, Hyeonju Ahn, Se-Young Kim, Yoon-Mo Kang, Seoae Cho, Heebal Kim

**Affiliations:** 1 C&K genomics, Seoul National University Research Park, Seoul 151-919, Republic of Korea; 2 Interdisciplinary Program in Bioinformatics, Seoul National University, Seoul 151-742, Republic of Korea; 3 Laboratory of Developmental Genetics, Department of Biomedical Sciences, Inha University School of Medicine, Incheon 22212, Republic of Korea; 4 Department of Agricultural, Food and Nutritional Science, University of Alberta, Edmonton, Alberta T6G 2P5, Canada; 5 Department of Agricultural Biotechnology and Research Institute of Agriculture and Life Sciences, Seoul National University, Seoul 151-921, Republic of Korea; 6 R&D center, CTCBIO, Inc. 38, Hyundaikia-ro, Paltan-myeon, Hwaseong-si, Gyeonggi-do, 18576, Republic of Korea

## Abstract

Results of recent studies on gut microbiota have suggested that obesogenic bacteria exacerbate obesity and metabolic dysfunction in the host when fed a high fat diet (HFD). In order to explore obesity-associated bacterial candidates and their response to diet, the composition of faecal bacterial communities was investigated by analyzing 16S rRNA gene sequences in mice. Dietary intervention with probiotics and *Garcinia cambogia* extract attenuated weight gain and adipocyte size in HFD-fed mice. To identify obesity-causative microbiota, two statistical analyses were performed. Forty-eight bacterial species were found to overlap between the two analyses, indicating the commonly identified species as diet-driven and obesity-associated, which would make them strong candidates for host-microbiome interaction on obesity. Finally, correlation based network analysis between diet, microbe, and host revealed that *Clostridium aminophilum*, a hyper-ammonia-producing bacterium, was highly correlated with obesity phenotypes and other associated bacteria, and shown to be suppressed by the combination of probiotics and *Garcinia cambogia* extract. Results of the present study suggest that probiotics and *Garcinia cambogia* extract alleviate weight gain and adiposity, in part via differentially modulating the composition of gut microbiota in HFD fed mice.

Obesity is a rapidly growing global health epidemic which confers great risk for the development of serious health issues such as diabetes, cardiovascular disease, and cancer^1^. Host-microbe studies unveiled the role of gut microbiota modifying immune responses^2^, inflammation and insulin resistance in the development of diet-induced obesity in animals^3^. Recent studies which involved transplanting faecal microbiota from obese humans to germ-free mice revealed that the gut microbiota itself may induce obesity^2^,^3^. Recent findings in the proportion of two major bacterial phyla (*Firmicutes* and *Bacteriodetes*) and their ratio between obese and lean samples (in humans and animals)^4^,^5^ have promoted the investigation of microbial candidates associated with obesity. Although growing evidence suggests positively or negatively obesity-associated microbes at various taxa^6^, only a few species have been well studied and understood^6^,^7^,^8^,^9^.

During the past decade, many researchers have focused on studying *Firmicutes* and its characteristic of increasing the capacity of energy harvest from indigestible sources in diet^5^,^10^,^11^, mainly due to carbohydrate metabolism processes. However, recent studies have observed that a western-style diet increases gut microbial amino acid metabolism in humans and animals^8^,^12^. High-fat feeding particularly increases microbial proteins of amino acid metabolism, which leads to the exceeding changes of gut microbial community composition in mice^12^. Short-term consumption of an animal-based diet compared to a plant-based diet significantly increases the faecal concentration of branched short chain fatty acids such as isovalerate and isobutyrate from amino acid fermentation in humans, indicating a quick shift between carbohydrate and amino acid fermentation in gut microbiota^8^.

Previous studies have indicated that dietary intervention such as probiotics and polyphenol-rich fruit extracts changes the composition of gut microbiota, and reduces visceral adiposity and obesity in animals^13^,^14^,^15^,^16^. Probiotics change the proportions of metabolic syndrome-related phylotypes in high fat fed mice, which differentially influences gut microbiota^14^. *Garcinia cambogia* extract containing hydroxycitric acid, known as an inhibitor of ATP-citrate-lyase in fatty acid biosynthesis, is polyphenol-rich and shows antibacterial and antioxidant properties.

In this study, we aimed to investigate which gut microbes respond to dietary intervention such as high fat, probiotics, and *Garcinia cambogia* extract, as well as whether they are associated with obesity phenotypes in diet-induced obese mice. After 9 weeks of dietary intervention, three types of metagenome analysis on faecal microbial communities were performed to detect obesity-causative microbes responding to diet: 1) identification of differentially abundant microbiota (DAM); 2) identification of obesity trait-associated microbiota (TAM); and 3) network analysis in order to consider comprehensive information underlying gut microbiome features of diet-microbe interaction, host-microbe interaction, microbe-microbe interaction, and finally all combined interactions.

## Results

### Adipocyte sizes sensitively respond to a high-fat diet

**Figure 1:**
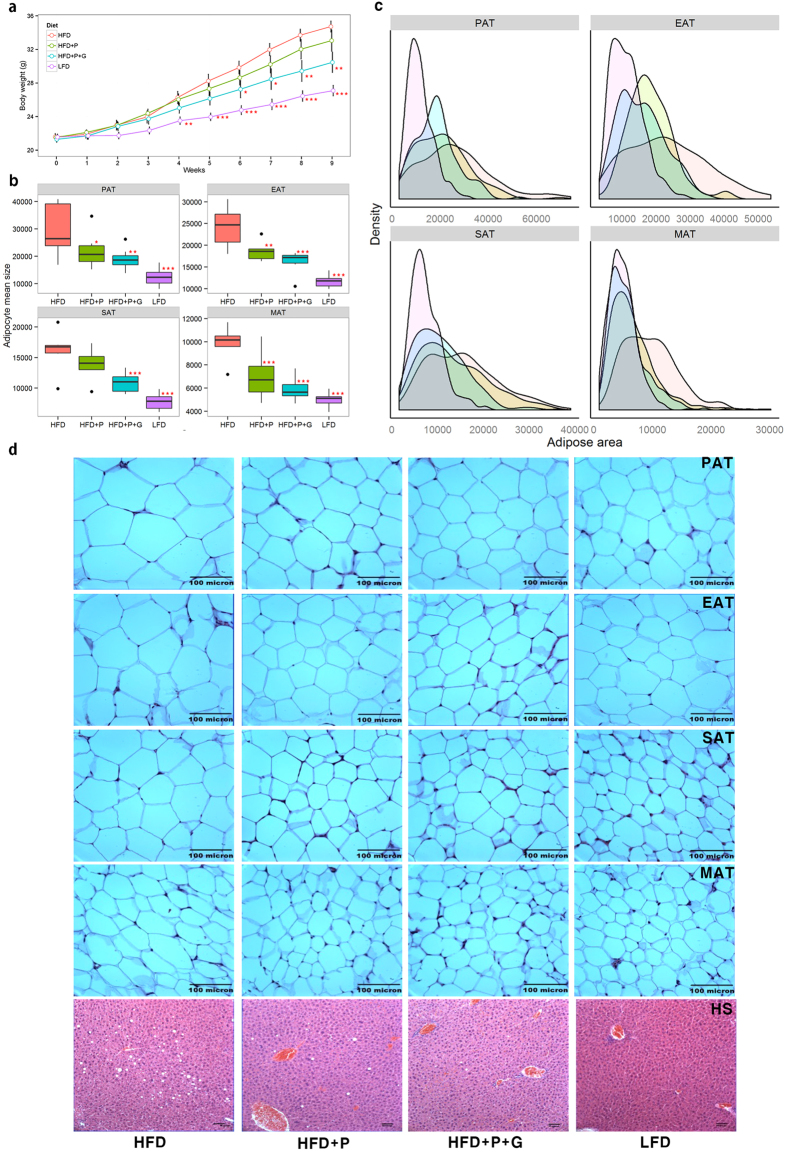
Dietary intervention affects adipocyte size as well as body weight in mice. Dietary groups were represented as different colors (Red: a high fat diet (HFD), Green: a HFD with 500 mg/kg BW of a probiotics mixture (HFD + P), Blue: a HFD with P + 1,000 mg/kg BW of *Garcinia cambogia* (HFD + P + G), and Purple: a low fat diet (LFD)) in all plots. Male C57BL/6J mice were fed one of four diets for 9 weeks (n = 7). (**a**) Body weight curves; Pairwise t-test was employed for significance test from HFD-fed animals (*P < 0.05, **P < 0.01, ***P < 0.001). (**b**) Different adipocyte mean sizes of four fat tissues such as epididymal adipocyte tissue (EAT), mesenteric adipocyte tissue (MAT), perirenal adipocyte tissue (PAT) and subcutaneous adipocyte tissue (SAT) responding to diet. (**c**) Density plots of adipocyte size in fat tissues. Y and X-axis are frequency and adipocyte size, respectively. (**d**) Representative adipose and liver tissue-staining images of four fat pads in mice. From top to bottom, PAT, EAT, SAT, MAT, and hepatic steatosis (HS), respectively. Black-bar is a barometer, which represents 100 microns. A pattern of gradually decreasing adipocyte size in four adipose tissues as well as fat granules in the liver tissue was observed. Data are represented as the mean ± SEM. Pairwise t-test was employed for significance test from HFD-fed animals (*P < 0.05, **P < 0.01, **P < 0.001).

To evaluate obesity traits responding to diet, male C57BL/6J mice were fed a low-fat diet (LFD), HFD, HFD with probiotics (HFD + P) and HFD with a combination of probiotics and *Garcinia cambogia* extract (HFD + P + G) for 9 weeks, respectively (diet information provided in Table S1, n = 7 for each treatment). Body weight was significantly lower in LFD mice after 4 weeks and in HFD + P + G mice after 6 weeks compared to HFD mice at 5% significance level (Fig. 1a and Table S2). Body weight gain was 58%, 13%, 31% lower in LFD mice, HFD + P mice, and HFD + P + G mice, respectively, compared to HFD mice (Table S3). All fat pad mass proportions to body weight in LFD were lower compared to HFD mice (Table S3). The mean sizes of adipocytes from perirenal, epididymal, and mesenteric adipose tissues (PAT, EAT, and MAT, respectively) were lower in HFD + P mice than those from HFD mice (Fig. 1b), except for subcutaneous (SAT) adipocyte. The density plots of adipocyte sizes showed a similar pattern, with mean values arranged in order from LFD to HFD groups (Fig. 1c). Adipocyte sizes of each mouse showed individual variability responding to diet which we determine, in part, as a result of gut microbiota in this study (Fig. S1a). Moreover, the box-plot patterns were very similar between weight gain and adipocyte mean sizes (Fig. 1b and Fig. S1b), which was concordant with representative tissue-staining images, presenting four kinds of adipocyte sizes and hepatic steatosis gradually increased from LFD to HFD (Fig. 1d).

### Change of serum biochemical parameters for protein metabolism in response to diet

The serum biochemical parameters were further analyzed to investigate host metabolism in response to diet. Blood urea nitrogen (BUN) and creatinine levels in the serum of HFD and HFD + P mice were found to be significantly lower than those in LFD and HDP + P + G mice (P < 0.001 and P < 0.01, respectively, Fig. S1c). Spearman correlation coefficients among diet, obesity traits and protein metabolism showed that diet order ascending from HFD to LFD had a negative correlation with obesity traits, particularly EAT adipocyte mean size (Correlation coefficient and P-value are −0.89 and 3.65E-10, respectively), and a positive correlation with serum BUN (0.65 and 1.99E-4) and creatinine (0.76 and 2.74E-06) (Fig. S1d). BUN and creatinine were negatively correlated with obesity traits, particularly with EAT adipocyte mean size (−0.66 [1.35E-04] and −0.65 [1.82E-04], respectively). Through correlation analysis, we observed strong linear relationships among diet, obesity traits and protein metabolism.

### Characterizing changes in microbial abundance in response to diet

Differentially abundant microbiota (DAM) analysis of 494 annotated operational taxonomic units (OTUs) at the species level was performed to characterize the changes of microbial species in response to diet (Dataset S1). Four types of comparisons were performed: a multi-group test (*H*_1_: at least one group ≠ 0 in the four dietary ingredients), HFD vs HFD + P, HFD vs HFD + P + G, and HFD vs LFD (descripted in Supplementary Methods). Of these statistical tests, we focused primarily on the result of the multi-group test given our primary goal of detecting causative bacterial species for obesity-related traits in response to diet. As a result, 266 significant differentially abundant species were identified with false discovery rate (FDR) adjusted P-value < 0.05. The distribution of significantly detected species at the phylum level revealed that most of the annotated phylum was *Firmicutes* (67%) followed by *Bacteroidetes* (14.11%) *Actinobacteria* (10.37%), *Proteobacteria* (4.15%), and others (<1.66%) (Fig. S2a). The hierarchical clustering analysis of 266 species showed that the faecal samples were clearly separated based on diet (Fig. S2b) except two samples (one HFD + P + G and one HFD + P), confirming that significantly abundant species were strongly influenced by diet. The number of significantly abundant species was highest in LFD (141 species), followed by HFD + P + G (112 species) and HFD + P (48 species) compared with the HFD group (Fig. S2c and Dataset S1). The heatmap of the relative abundances of 50 bacterial species (mostly affiliated with three phyla such as *Firmicutes, Bacteroidetes* and *Actinobacteria*) showed that the maximum median proportion (MMP) among dietary groups was more than a thousandth (MMP > 0.1%) and also revealed dietary dependent patterns (Fig. S2a and Dataset S1). The microbial species that are susceptible to diet, showed four representative patterns, which are visualized as box-plots (Fig. S2d). The first is linearly decreasing from HFD to LFD such as *C. aminophilum* and *Oxobacter pfennigii.* The second is highly abundant in HFD groups such as *Clostridium propionicum, Clostridium scindens* and *Eubacterium fissicatena.* The third is highly abundant in LFD group such as *Ruminococcus bromii* and *rumen bacterium NK4A66.* The fourth is highly abundant or suppressed in HFD + P and HFD + P + G groups such as *Bifidobacterium breve, Bifidobacterium bifidum, Lactobacillus plantarum, Lactococcus garvieae* and *Lactobacillus johnsonii*. Particularly, *C. aminophilum* increased 32-fold in HFD mice comparing to LFD mice followed by a 12-fold increase of *C. propionicum*, which accounted for the highest proportion (MMP = 5.3%) among *Clostridium* species. Contrastingly, the abundance of *R. bromii*, a causative starch-degrading bacterium in human colon^17^, and unclassified *rumen bacterium NK4A66* decreased (1.20E-5 and 6.99E-10, respectively). In addition, we observed elevated proportions of bile acid transforming^18^ (*C. scindens*) and acetogenic^19^,^20^ (*O. pfennigii* and *E. fissicatena*) bacteria in HFD mice compared to LFD mice.

### Dietary intervention affects the proportion of *Bifidobacteria* and high fat-induced bacteria

**Figure 2:**
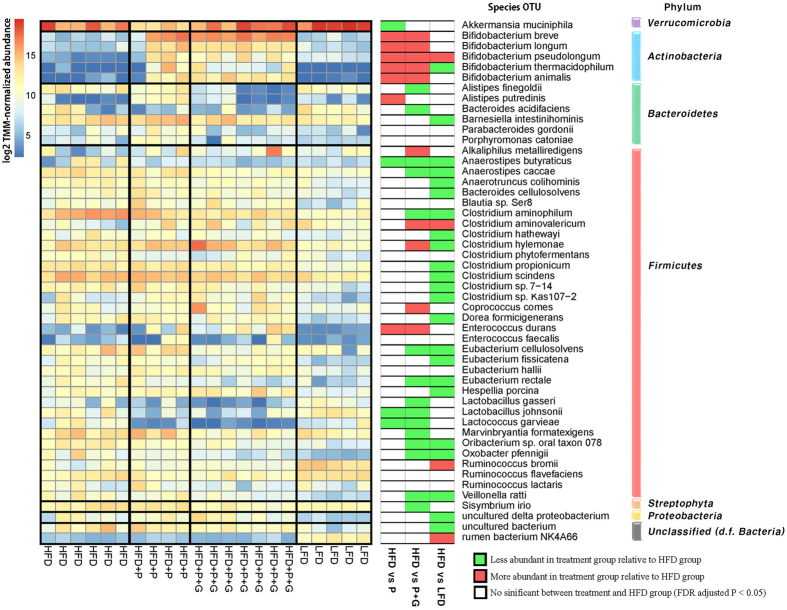
Dietary intervention induces relative abundance alteration in gut microbial species. The left heatmap illustrates 50 significantly detected species in the multi-group test (FDR adjusted P < 0.05) and maximum median proportion more than a thousandth responding to diet. Species abundance is represented as log2-counts per million reads (log-CPM). The second heatmap represents results of two group comparison based on the HFD group. The phylum level taxonomy is given on the right.

Administration of a probiotic mixture (*B. breve, B. bifidum L. plantarum, L. acidophilus and Enterococcus faecium)* to HFD mice increased the abundance of 10 species out of the total 16 surveyed *Bifidobacteria* species including the increases in administrated *B. breve* and *B. bifidum* (Fig. 2; See also Dataset S1). However, only 2 out of 34 *Lacotobacillus* species (*L. plantarum* and *L. pentosus*) increased after the probiotic treatment, and the abundance of *L. johnsonii*, the most abundant commensal species in the genus *Lactobacillus* (MMP = 0.6%), was reduced due to the external probiotics. In addition, administrated *Bifidobacteria* decreased proportions of *A. butyraticus* and *L. garvieae,* (MMP = 0.3% and 0.1%, respectively). The combination of probiotics and fruit extract decreased the proportion of high fat-induced bacteria such as *C. aminophilum* and *O. pfennigii*. Combined dietary intervention (3.42E-8) more effectively suppressed *C. aminophilum* than a probiotics mixture (0.3) compared to the HFD group; a similar trend was observed for *O. pfennigii*.

### Characterizing obesity trait associated microbiota

**Figure 3:**
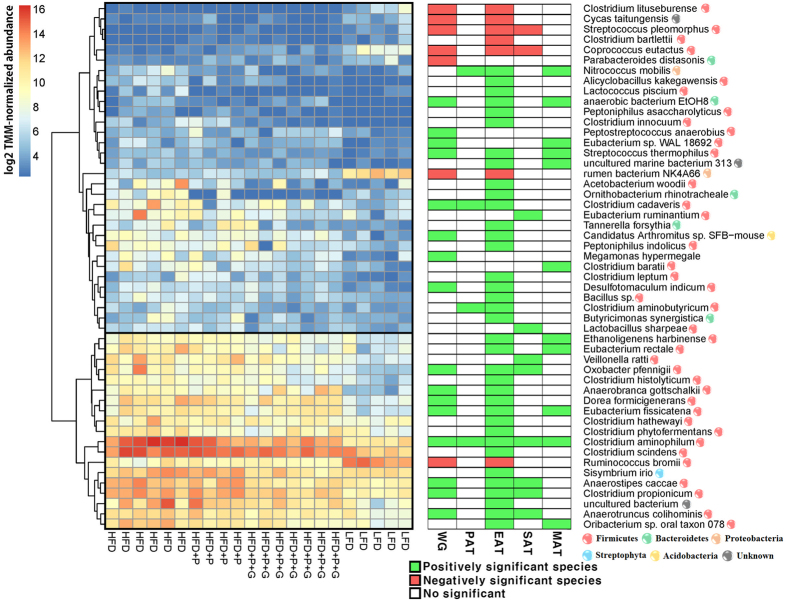
Obesity traits are highly associated with gut microbial species. The left heatmap illustrates 48 overlapped species, which are significantly detected in DAM and TAM analyses. Species abundance is represented as log-CPM values. The second heatmap represents test results in TAM analysis with five response variables (FDR adjusted P-value < 0.1).

A linear regression model was employed to identify obesity trait-associated microbiota (TAM), with five obesity traits including weight gain and four types of adipocyte measures (additional description available in Supplementary Methods). As a result, 62 bacterial species were significantly (FDR adjusted P < 0.1) associated with five obesity traits (Fig. S3a and Table S4). Among those, 51 species were found to overlap between DAM and TAM analyses (Fig. S3b), indicating these commonly identified species not only respond to diet but are also associated with obesity traits. 41 out of 51 bacterial species were highly correlated with EAT adipocyte mean size (Fig. S3a). To identify the relationship between EAT adipocyte mean size and bacterial abundance, the top 15 significantly associated bacterial species were visualized as a fitted line plot. Bacterial species detected by TAM analysis show strong linear relationship (0.36 ≤ R^2^ ≤ 0.75) (Fig. S3c). Furthermore, the hierarchical clustering analysis of the 48 commonly identified species (excluding two unknown and one plant annotations) displayed two separate groups (Fig. 3), with the lower group having higher species proportions. When the bacterial species were annotated to their corresponding phyla, they were mostly *Firmicutes*, while only 8 species belonged to *Bacteroidetes, Proteobacteria, Streptophyta* and *Acidobacteria. C. aminophilum* was the only species associated with all surveyed obesity traits and the most significantly associated species (FDR adjusted P value = 9.19E-05 in EAT adipocyte mean size). The abundance of *C. aminophilum* also had a negative association with host parameters of protein metabolism such as serum BUN and creatinine (Fig. S3d). In addition, *C. propionicum*, a propionic acid producing AP bacterium (28), was positively associated with three obesity traits, and negatively correlated with serum BUN and creatinine.

### Correlation based network analysis using relative abundances of significantly detected microbiomes in DAM and TAM with obesity-related traits

**Figure 4:**
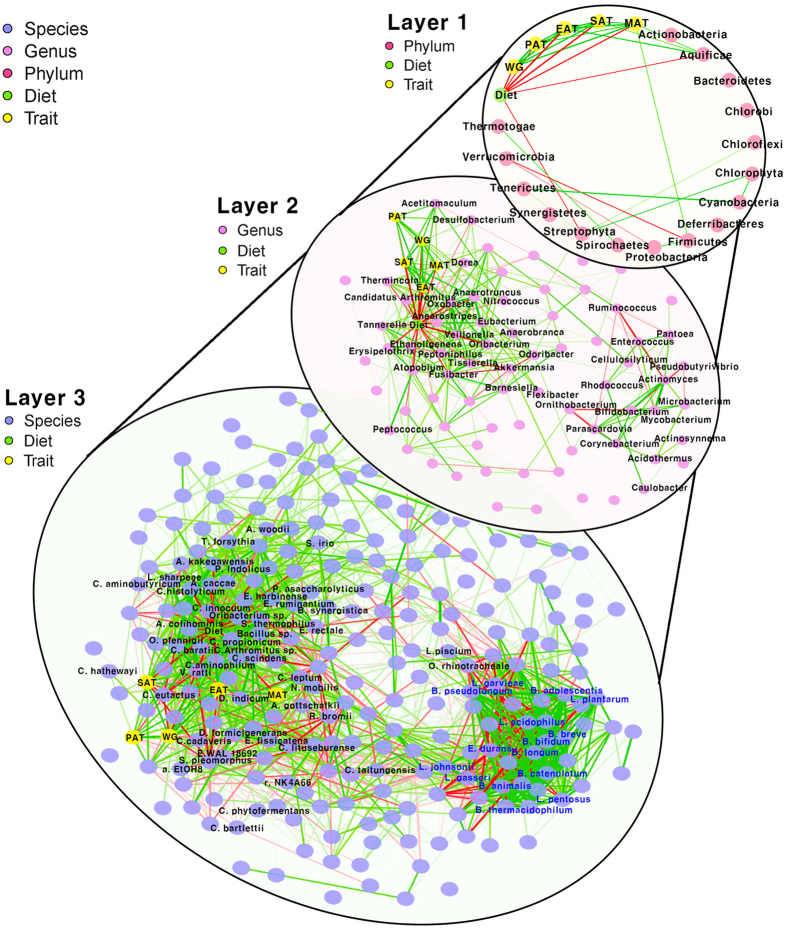
Correlation based network using relative abundance of gut microbes on multi-level taxa. Each node represents each phylotype in the different taxonomic layers (plylum, genus, and species). The green and red edge represents the direction of Spearman correlation; positive and negative, respectively. In addition, line width indicates the strength of correlation. Only the significant edges are drawn in the network using the Spearman correlation test (FDR adjusted P < 0.01). In Layer 1, relationships between the relative abundance of phyla and traits including diet are represented with full labelling. Second and third layers represent the relationships in genus and species level, respectively. In Layer 3, 48 significantly detected species from the DAM and TAM analyses (black texts), and administrated probiotics such as *B. breve, B. bifidum, L. plantarum, and L. acidophilus* with other surrounding commensal bacteria (blue texts) are labeled.

Correlation based network analysis was performed using multi-level taxa to visually investigate the microbe-microbe relationships in addition to the afore-mentioned diet-microbe and host-microbe relationships (Fig. 4). As a result, network plots exhibit a highly complex structure despite the strict P-value cut-off for significant relationships (Spearman correlation test with FDR adjusted P < 0.01) at each taxa level. Of the many relationships, two representative sub-networks were observed at the genus and species levels. The first sub-network included diet, obesity traits, and most of the commonly identified bacterial species between DAM and TAM analyses (Fig. 4, Layer 3) with nodes of commonly identified species highly correlated with each other. Particularly, *C. aminophilum* was highly correlated with commonly identified species (0.334 ≤ *R*^2^ ≤ 0.824) and the average Spearman correlation was 0.594 (Table S4), indicating that *C. aminophilum* is a strong candidate for diet driven obesity. In the second sub-network, administrated probiotics (*B. breve, B. bifidum L. plantarum* and *L. acidophilus)* formed complex nodes with surrounding commensal *Bifiodobacteiria, Lactobacilli, L. garvieae* and *E. durans,* indicating an important role of external probiotics to overall bacterial abundance.

## Discussion

In this study, we selected probiotics and *Garcinia cambogia* extract for dietary intervention, which have been reported to support weight management in humans. Some probiotic strains and probiotic mixtures were shown to be effective in weight loss, visceral adiposity, and related metabolic syndrome in animals^13^,^14^,^21^. *Garcinia cambogia* extract containing hydroxycitric acid (HCA), known as an inhibitor of ATP-citrate-lyase in fatty acid biosynthesis, attenuated obesity traits in humans and animals^22^,^23^. However, these interventions are controversial in their efficacy in humans^24^,^25^. We presume that probiotic strains used in this study would be integrated into gut microbiota, promoting its composition to the direction of attenuation in obesity development, in synergy with host metabolism influenced by *Garcinia cambogia* extract.

The combination of these two supplements reduced weight gain and adipocyte mean sizes, but the use of probiotics alone attenuated adipocyte mean sizes only. Statistically, adipocyte mean size is generally preferred over weight gain because of its robustness and sentiveness^26^. In addition, we presumed that associated microbes may be associated more with metabolically active EAT (equivalent to human VAT) than SAT^27^. Concordantly, our TAM results support that epididymal adipocyte mean size is more sensitive than other measurements for the degree of obesity (Weight gain, PAT, SAT, and MAT), as shown in Fig. S3. However, further research is required to clearly understand the degree of association between microbes and different adipose depots.

The main objective of this study is to determine effectively whether combined supplements substantially modulate obesity-associated microbes characterized in mice with diet-induced obesity. To achieve this objective, two types of statistical tests were jointly employed to identify obesity-causative microbes (Fig. S4; see also supplementary methods). Recently, metagenome analysis has focused solely on the detection of DAM in given experimental conditions. However, only comparing the relative abundance is a limited approach in that diet-induced gut microbes may not be obesity-associated microbes. Instead, they can be consequences of microbial fermentation responding to their preferred dietary ingredients. Given the situation, we presumed that diet-driven causative microbial candidates should be DAM responding to diet as well as significantly associated with obesity phenotypes and various recent attempts on obesity-associated microbes detection using linear regression models have been successfully reported^28^,^29^,^30^. For these reasons, a two-stage statistical test on DAM and TAM was simultaneously considered, which was suitable for this study. Considering our objective of detecting causative microbes, of changing obesity traits responding to dietary intervention, the candidate microbes should not only be differentially abundant in response to diet, but also significantly associated with obesity-related traits. As far as we know, although this approach has been previously used in transcriptomic studies, this is the first attempt to use it in the microbiome study^31^. The progression of method development in this field is similar to that of transcriptome analysis such as RNA-seq and microarray. Therefore, a well-developed statistical methodology for identifying causal genes could be applied to the microbial community analysis with minor adjustments^32^. Especially, application of network analysis is important in gut microbiota research because a microbial community is composed of complex relationships comparable to the transcriptome. While several metagenome studies have been performed with network analysis and pivotal OTUs in the microbe-microbe relationships have been successfully detected, complex causality relationships (diet-microbe, host-microbe, and microbe-microbe) were utilized in this study for the first time (Fig. 4).

A greater number of gut microbes were affected by LFD (141 species), HFD + P + G (112 species) and HFD + P (48 species), in order, compared to the HFD feeding. HFD feeding increased the abundance of many species affiliated with the phylum *Firmicutes* (Fig. 2), which is in agreement with other studies^5^,^9^,^14^. Pathophysiological evidence supports our finding that HFD-driven microbiota increases gut permeability and metabolic endotoxemia resulting in chronic inflammation on adipocytes; as a result, exacerbates obesity and its metabolic syndrome^33^,^34^,^35^. However, while probiotics promoted the abundance of other commensal *Bifidobacteria*, the probiotic effect on abundance was minimal to commensal *Lactobacilli.*

We observed that the combination of probiotics and *Garcinia cambogia* extract highly changed the gut microbial community compared to the effect of probiotics only. Because many researchers have focused on the role of HCA for the control of host adiposity in the point of fatty acid metabolism, not much is known about the effect of *Garcinia cambogia* extract on gut microbiota and its modulating potential. Taking into account that *Garcinia cambogia* extract is polyphenol-rich, performs antibacterial and antioxidant functions^36^, and ameliorates gastric ulcer^37^ and colitis in rats^38^, we hypothesized that it could modulate gut microbiota in the mode of action of polyphenols proposed by other *in vitro* and *in vivo* studies^15^,^16^,^39^,^40^. Our DAM results may explain, in part, the controversy on the efficacy of *Garcinia cambogia* extract on weight loss and adiposity in humans^23^,^24^,^41^, affected by the compositional variation of individual gut microbiota^42^. Our study selected probiotics and *Garcinia cambogia* extract, for dietary intervention showed increased abundance of *Bifidobacteria* and decreased hyper-ammonia-producing (HAP) bacteria, suggesting the potential to reduce obesity through manipulation of gut microbiota under high-fat diet. This is the first study to reveal that dietary interventions such as additional probiotics or polyphenol-rich fruit extract are plausible candidates to suppress the blooming of HAP and analogs in the gut microbial community in humans.

In this study, a HFD + G and P + G groups were not considered,therefore the individual effect of *Garcinia cambogia* extract cannot be characterized. Further studies are needed to determine whether the sole use of *Garcinia cambogia* extract effectively changes gut microbial community and how much it contributes to alleviating obesity development via microbial modulation comparing to the known role of down-regulating fatty acid synthesis in the host.

As a result of a two-stage statistical test on 16S rRNA taxonomic data, 48 bacterial species were identified as putative candidates for host-microbe interaction in the development of obesity. Of note, *C. aminophilum*, the member of HAP bacteria and non-saccharolytic obligate amino acid fermenters, was highly abundant, and the most significantly associated with weight gain and adipocyte sizes in mice, which was suppressed by the combined use of probiotics and *Garcinia cambogia* extract in HFD mice. The typical HFD mimicking a Western-style diet (Table S1) in our study contains only 20% of energy from carbohydrates such as sucrose (7%) and maltodextrin (12%) quickly absorbed by upper small intestine^43^,^44^, which may induce the depletion of carbohydrates in distal ileum and colon where the majority number of gut microbes reside^45^. We speculate that such severe depletion shifted the dominant bacteria from carbohydrate to amino acid fermenters such as *C. aminophilum* and *C. propionicum* in HFD mice. The changes in microbial transcriptome and metabolites on amino acid and carbohydrate metabolism in Western-style diet reported by other gut microbiome studies^8^,^12^ are in agreement with the blooming of amino acid fermenters and the withering of *R. bromii* in HFD mice. In cattle and dairy cows, ammonia-producing (AP) bacteria have been intensively investigated to better understand excess ammonia production and amino acid wasting for meat and milk production^46^. Since the late 1980s, researchers have reported that specific AP bacterial species with very high ammonia producing activity (>300 nmol NH_3_ mg^−1^ protein min^−1^) mostly affiliated with the genus *Clostridium, Eubacterium, Peptostreptococcus* in the phylum *Firmicutes*^47^,^48^,^49^,^50^,^51^. These HAP bacteria play a causative role in influencing microbial nitrogen metabolism by producing NH_3_ up to 20-fold faster than other AP bacteria^48^,^50^,^51^,^52^. Interestingly, the abundance of *C. aminophilum* and *C. propionicum* had a negative association with serum blood urea nitrogen and creatinine (Fig. S3d). Further research is required to elucidate why these bacteria are associated with host protein metabolites and whether they are involved in the development of obesity.

The two-stage test also revealed increased abundance of a bile acid transformer and other bacterial groups which responded to diet and were associated with obesity traits. The abundance of *C. scindens*, which is responsible for converting primary bile acids to secondary deteriorated bile acids^18^, was increased 7-fold after HFD (Fig. S2). The abundance of acetogens such as *O. pfennigii* and *Acetobacterium woodii* in the class *Clostridia* were positively associated with obesity traits (Fig. S3c), suggesting their role may be related to efficiency for energy harvest in the large bowel^53^. *O. pfennigi*, previously classified as *Clostridium pfennigi*, produces acetate and butyrate using CO^19^, and *A. woodii*, homoacetogens, produces acetate only using H_2_ gas and CO_2_, eliminating accumulated H_2_ gas for gut microbiota to consistently ferment substrates^54^. *Candidatus* Arthromitus *sp*. SFB-mouse, nonpathogenic segmented filamentous bacteria related with induction of strong host immune responses to intestinal epithelial cell^55^, showed a positive linear relationship with EAT adipocyte mean size (Fig. S3c). Further research using molecular and pathophysiological approaches is needed to validate our results based on correlative analyses.

In conclusion, various attempts to understand the dynamic relationship between gut microbiota and obesity-related traits responding to diet revealed which gut microbes are associated with obesity phenotypes. First, obesity-related 48 species candidates were identified from DAM and TAM analyses. Also, the result of network analysis provides the blueprint to explain the complex relationship among microbes, diet, and host’ phenotypes, which possibly explains the effect of probiotics and *Garcinia cambogia* extract on gut microbiota features associated with obesity traits. Employed methodology and following results could be applied to further research the taxonomic signature of obesity to develop dietary intervention and treatments supporting weight management in overweight and obese humans.

## Materials and Methods

### Animals and diets

Male C57BL/6J mice (6 weeks old, n = 7 per group) were purchased from Orient Bio Inc. (Sungnam, South Korea). Mice were housed in autoclaved cages (three or four mice per cage) in a pathogen-free room with the temperature and relative humidity at 22 ± 2 °C and 50 ± 10%, respectively, under a 12-h light-dark cycle. The low fat (LFD; fat 10 kcal%, carbohydrate 70 kcal%, protein 20kcal%; D12450B) and high fat (HFD; fat 60 kcal%, carbohydrate 20 kcal%, protein 20 kcal%; D12492) diets were obtained from Research Diets Inc., New Brunswick, NJ (Table S1). All animal studies were performed in accordance with the Korean Food and Drug Administration (KFDA) guidelines. The experimental procedures were reviewed and approved by the Institutional Animal Care and Use Committee of the INHA University (No. 140728-320).

The probiotic mixture (1 × 10^11 ^CFU/g) based on Double Impact^®^ formula (DI formula) and *Garcinia camb*ogia extract (HCA content is over 60%) originated from the INDFRAG (India); Product name is garcinia cambogia husk extract PE65, was obtained from CTC Bio Co., Ltd., Seoul, Korea. The probiotic mixture consisted of two strains of *Bifidobacteria (B. breve* LMC520 (2 × 10^10 ^CFU/g; 20%) and *B. bifidum* CBF0829 (2 × 10^10 ^CFU/g; 20%)), two strains of *Lactobacilli (L. acidophilus* CLB0413 (0.8 × 10^10 ^CFU/g; 8%) and *L. plantarum* CLP0611 (2.4 × 10^10 ^CFU/g; 24%)) and one strain of *Enterococcus faecium* CEF-1 (2.8 × 10^10 ^CFU/g; 28%). After a 1 week acclimatization period on LFD, mice were divided into four weight-matched groups: LFD group, HFD group, HFD + probiotics mixture (500 mg/kg body weight) (HFD + P) group, HFD + probiotics mixture (500 mg/kg body weight) and *Garcinia cambogia* extract (1,000 mg/kg body weight) (HFD + P + G) group. Probiotics and *Garcinia cambogia* extract (dissolved in PBS) were administrated by oral gavage, and LFD and HFD groups were treated with equal volumes of PBS for 9 weeks. Irradiated diets and water were fed ad libitum, and body weight and feed intake was recorded twice every week. At the end of the animal study, all the mice fasted overnight and were anesthetized with Zoletil^®^ and Rompun^®^. Blood was collected by cardiac puncture, and serum was isolated by centrifugation at 3,000 rpm for 10 min at 4 °C, and stored at −80 °C for further biochemical analyses. Mice were sacrificed by cervical dislocation after blood collection. Adipose tissues (perirenal, epididymal, subcutaneous, mesenteric) and liver were removed precisely, and then weighed.

### Histochemical staining and adipocyte surface area analysis

Adipose tissues (mesenteric, subcutaneous, epididymal, and perirenal) and liver tissues were fixed with 10% buffered neutral formalin, embedded in paraffin, sectioned at 4 *μ*m, stained with hematoxylin and eosin, viewed with Axioplan 2 imaging (CarlZeiss, Jena, Germany), and photographed at a final magnification of 400X. Adipocyte sizes were measured using Molecular Imaging software (Bruker Biospin, Woodbridge, CT, USA).

### Statistical analysis for mice experiment

Data is represented as the mean ± SEM. Adipocyte mean areas and body weight gain were analyzed using a pairwise t-test. Body weight gain curves were statistically compared by using a two-way repeated measures ANOVA. All statistical results were significantly considered at P-value < 0.05. Statistical analysis was performed by using R.

### Fecal DNA preparation and microbial community analysis

Fecal pellets were collected from individual mice in a sterile tube and immediately frozen by dry ice and stored at −80 °C. DNA was isolated using Epicentre DNA isolation kits (7 biological replicates in each dietary group). Approximately 900 ng of DNA was extracted from each sample. DNA quality was confirmed by Bioanalyzer using an Agilent RNA 6000 Pico Kit (Agilent, Santa Clara, CA). All samples from the reservoir were prepared using the 16S library preparation protocol and the Nextera XT DNA index kit (illumina, San Diego, CA) to target the V3-V4 variable regions of the 16S rRNA gene. Library quantification was measured by real-time PCR using the CFX96 real time system (BioRad, Hercules, CA). 22 from a total of 28 mice samples passed the experimental quality control, and six samples (2 LFD, 1 HFD, and 3 HFD + P samples) failed to generate amplicon because of the subject’s poor fecal state (diarrhea). Samples from the reservoir were loaded onto a MiSeq reagent catridge (illumina, San Diego, CA) and then onto the instrument. Automated cluster generation and 2 × 300 bp paired-end sequencing were performed. The resulting sequence reads were equally distributed across the samples.

### Pipeline for microbial community analysis

We employed Trimmomatic^56^ with the following options: PE -phred33 ILLUMINACLIP:TruSeq3-PE.fa:2:30:10 MINLEN:75 2 for removing adapter sequences on each read. After generating clean reads, we overlapped sequences using FLASH-1.2.11^57^ with the following options: –min-overlap 30 –max-overlap 200. These pre-processed sequences were uploaded to the MG-RAST server^58^ for calculation of microbioal features. Microbial features were extracted using the MG-RAST analysis tool.

### Statistical analysis for microbial community analysis

Multi-level taxonomic abundance was extracted using MG-RAST. A negative bionomical based generalized linear model (GLM) was used to detect differentially abundant microbiota (DAM). For consideration of different read production, the trimmed mean of M-values (TMM) normalization method was used. Finally, a simple linear regression model was used for detecting trait associated microbiota (TAM). All statistical test results were adjusted using the Benjamini-Hochberg method^59^ for correction of multiple testing errors, setting the FDR at 10%. A more detailed description of statistical methods is available in Supplementary Methods.

## Additional Information

**Accession codes:** The microbial sequencing data has been deposited in NCBI’s BioProject (PRJNA290695) and SRA sequence database (SRR2124868).

**How to cite this article**: Heo, J. *et al*. Gut microbiota Modulated by Probiotics and *Garcinia cambogia* Extract Correlate with Weight Gain and Adipocyte Sizes in High Fat-Fed Mice. *Sci. Rep.* **6**, 33566; doi: 10.1038/srep33566 (2016).

## Supplementary Material

Supplementary Information

Supplementary Dataset S1
