# Supplementary material for: Gut microbiota Modulated by Probiotics and Garcinia cambogia Extract Correlate with Weight Gain and Adipocyte Sizes in High Fat-Fed Mice
Source: Sci Rep. 2016 Sep 23;6:33566. doi: 10.1038/srep33566 (PMC5034228; doi:10.1038/srep33566)
Supplement: Supplementary Information [file srep33566-s1.pdf]

Gut microbiota Modulated by Probiotics and *Garcinia cambogia* Extract Correlate with Weight Gain and Adipocyte Sizes in High Fat-Fed Mice

Jaeyoung Heo[1*], Minseok Seo[1,2*], Hwanhee Park[3], Woon Kyu Lee[3], Le Luo Guan[4], Joon Yoon[2], Kelsey Caetano-Anolles[5], Hyeonju Ahn[5], Yoon-Mo Kang[6], Se-Young Kim[6], Seoae Cho[1§], Heebal Kim[1,2,5§]

[1]C&K genomics, Seoul National University Research Park, Seoul 151-919, Republic of Korea.

[2]Interdisciplinary Program in Bioinformatics, Seoul National University, Seoul 151-742, Republic of Korea.

[3]Laboratory of Developmental Genetics, Department of Biomedical Sciences, Inha University School of Medicine, Incheon 22212, Republic of Korea[4]Department of Agricultural, Food and Nutritional Science, University of Alberta, Edmonton, Alberta T6G 2P5, Canada.

[5]Department of Agricultural Biotechnology and Research Institute of Agriculture and Life Sciences, Seoul National University, Seoul 151-921, Republic of Korea.

[6]R&D center, CTCBIO, Inc. 38, Hyundaikia-ro, Paltan-myeon, Hwaseong-si, Gyeonggi-do, 18576, Republic of Korea

[¶] These authors contributed equally to this work
[§] Corresponding author

Heebal Kim : Tel: +82-2-880-4803, Fax: +82-2-883-8812 ; E-mail : heebal@snu.ac.kr
Seoae Cho : Tel: +82-2-876-8820, Fax: +82-2-876-8827 ; E-mail : seoae@cnkgenomic.com

Contents for supplementary materials

Fig. S1. Dietary intervention affects adipocyte size as well as body weight in mice. 3
Fig. S2. Significantly observed gut microbial OTUs from the DAM analysis.......... 4
Fig. S3. Significantly detected gut microbial OTUs from the TAM analysis. 5
Fig. S4. Schematic diagram of DAM and TAM analysis. 6

Table S1. Formula of low fat diet (LFD) and high fat diet (HFD). 7
Table S2. Dietary intervention effects on body weight in mice for 9 weeks (g). 8
Table S3. Dietary intervention effects on body weight gain and fat mass accumulation. 9
Table S4. Spearman correlation between C. aminophilum and other commonly identified bacteria from DAM and TAM analysis. 10

Method S1. Supplementary Methods 12

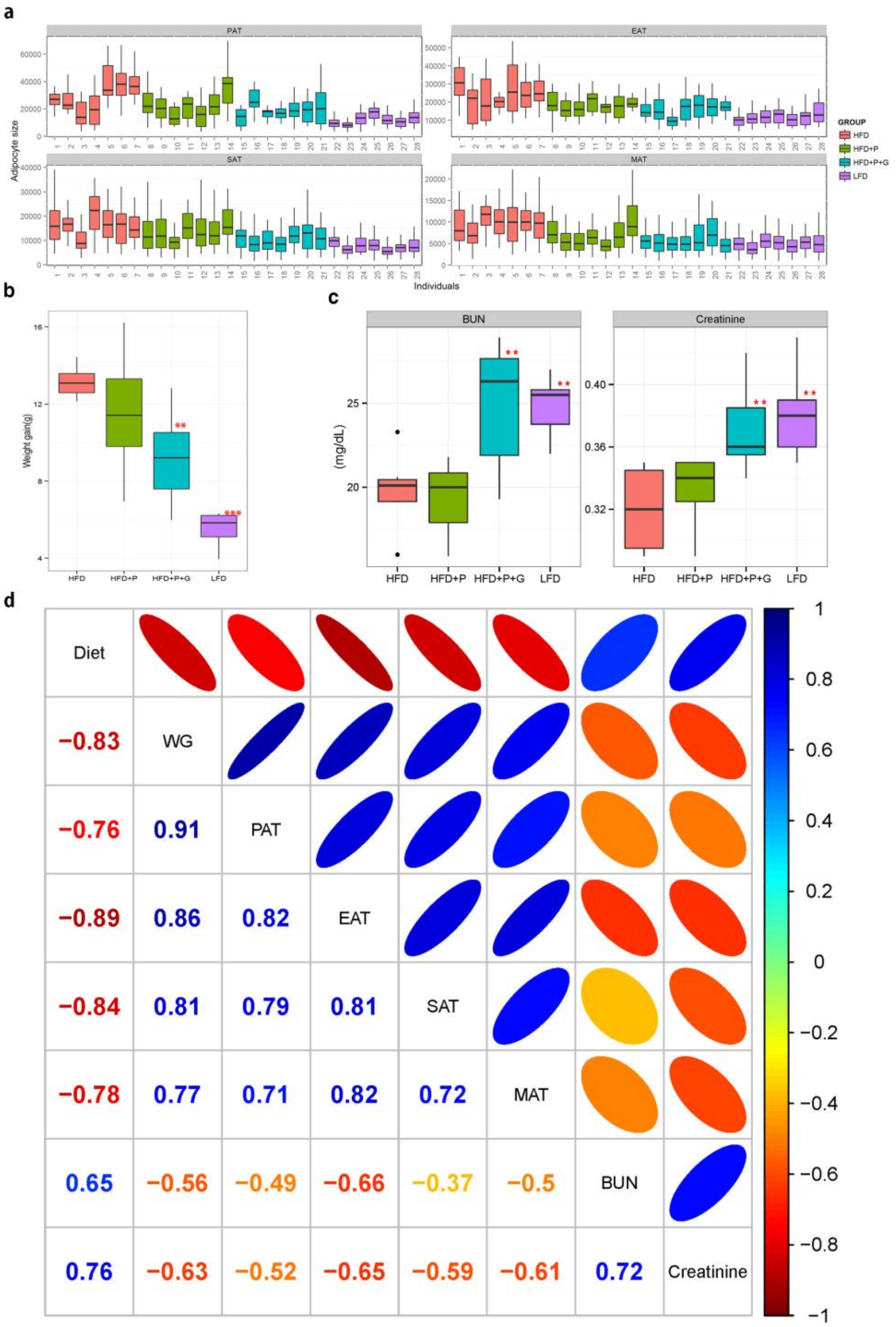

Fig. S1. Dietary intervention affects adipocyte size as well as body weight in mice.
(**a**) Individual level box-plot using repeatedly measured adipocyte size. (**b**) The box plot for weight gain responding to diet intervention (9 weeks). **(c)** Box-plots of serum blood urea nitrogen (BUN) and creatinine responding to diet (n=7). Data are represented as the mean ± SEM. Pairwise t-test was employed for significance test from HFD-fed animals (*P < 0.05, **P < 0.01, ***P < 0.001). **(d)** A correlation plot is composed of 8 variables including diet information (Diet), weight gain (WG), four adipocyte mean sizes (PAT, EAT, SAT, MAT), BUN and creatinine. Dietary information was codded in the following order (HFD = 1, HFD+P = 2, HFD+P+G = 3, and LFD = 4**).**

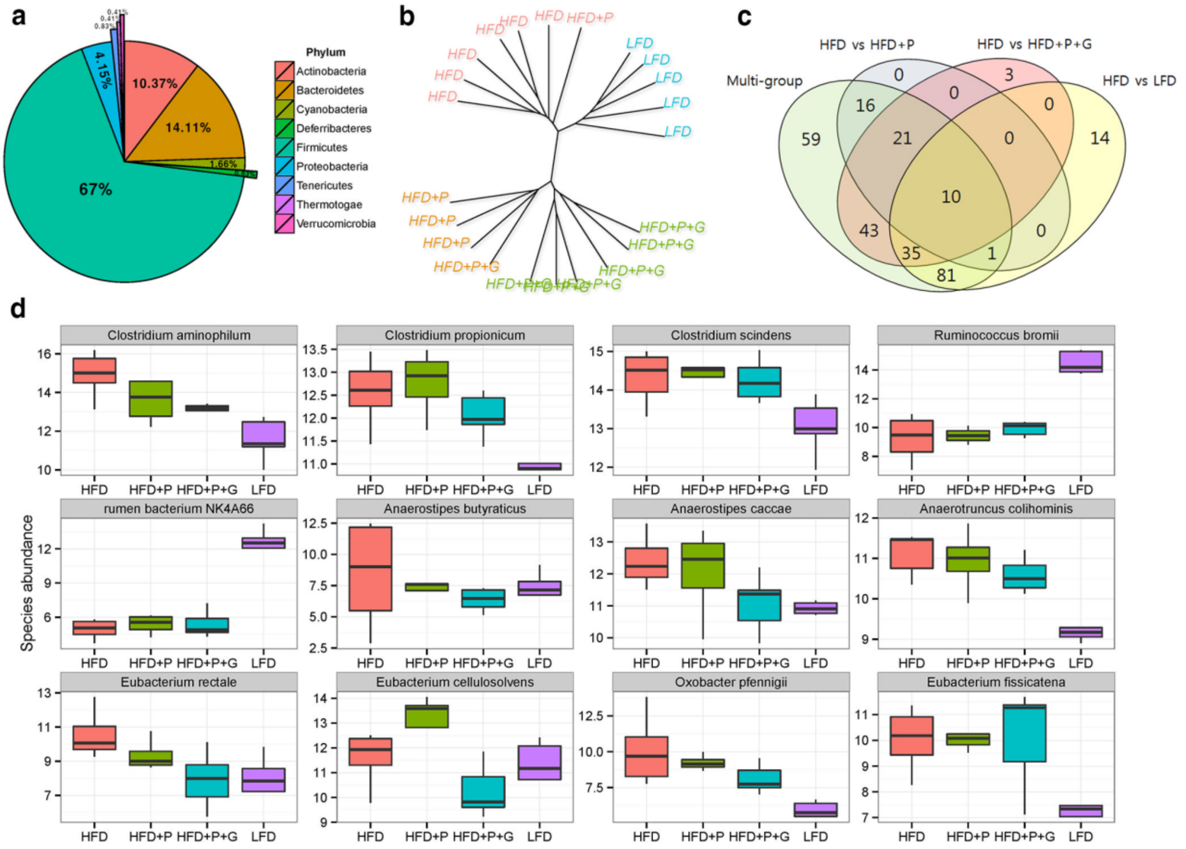

Fig. S2. Significantly observed gut microbial OTUs from the DAM analysis.
(a) Pie-chart of the phylum proportion from the 266 significantly detected OTUs at the species level in multi-group test and their annotated information in phylum level. Pie-charts depict mean standardized phylum abundance (% of total) responding to diet. Small proportioned phyla (<1%) were excluded in these pie charts. (b) Tree visualization based on the hierarchical clustering with cutting criteria; *k*=4, using 266 significantly detected species in multi-group test (FDR adjusted P < 0.05). (c) Venn diagram of detected species in four types of hypothesis tests: multi-group test, HFD vs HFD+P, HFD vs HFD+P+G, and HFD vs LFD group comparisons. (d) Box-plots of diet susceptible bacterial species.

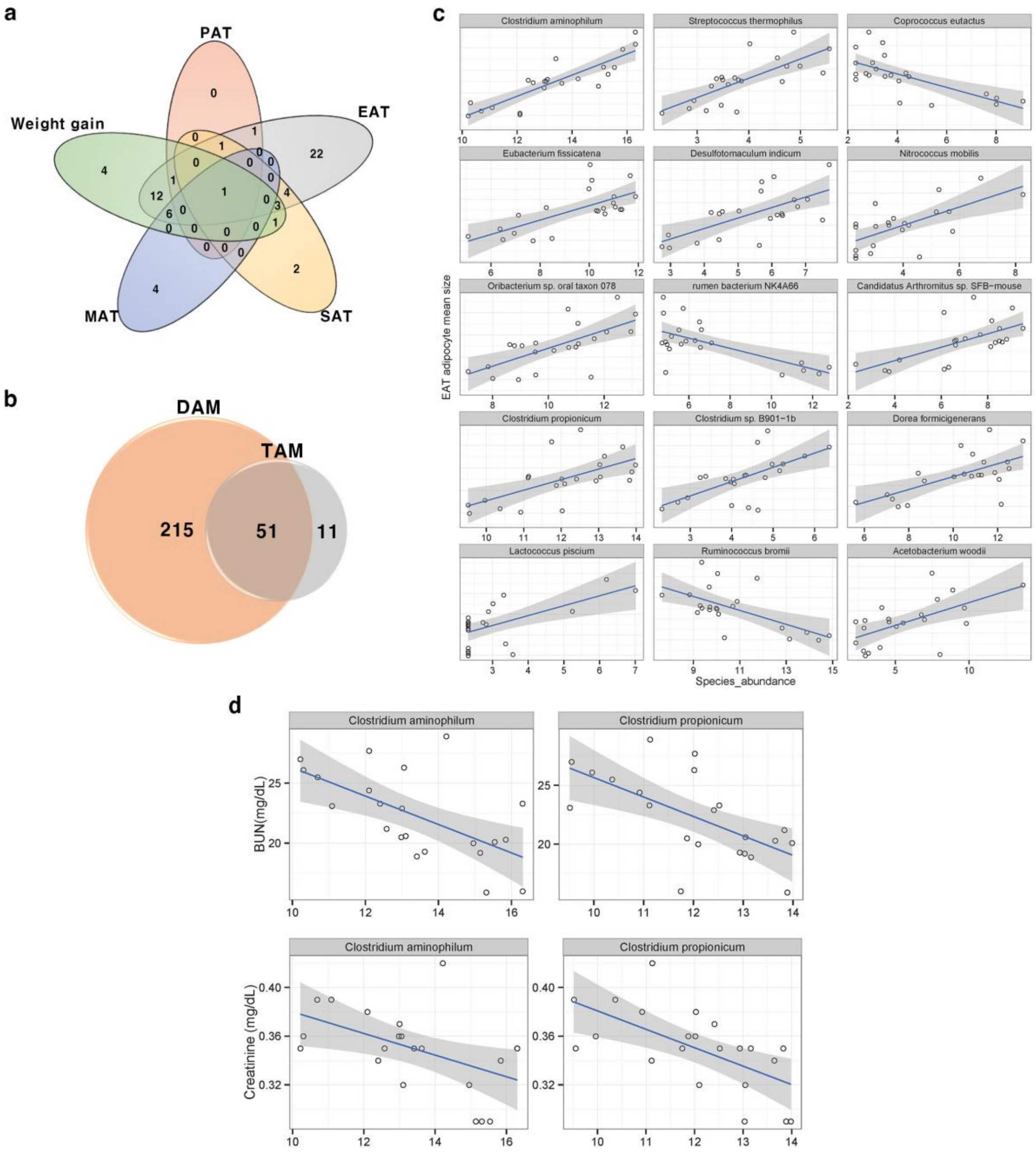

Fig. S3. Significantly detected gut microbial OTUs from the TAM analysis.
(a) Venn-diagram comparing significantly detected species numbers in TAM analysis between species abundance and five obesity traits such as weight gain (WG) and four kinds of adipocyte mean sizes (PAT, EAT, SAT and MAT), respectively. **(b)** Venn-diagram comparing significantly detected species between DAM and TAM analyses. **(c)** Scatter plots with fitted-line of linear regression using 15 significantly detected EAT associated species in TAM analysis with FDR adjusted P < 0.1. The blue line is fitted line in linear regression and gray region represents standard errors. **(d)** Scatter plots with fitted-line of linear regression. The blue line is fitted line in linear regression and gray region represents standard errors. Clostridium aminophilum and C. propionicum are significantly associated with BUN (P= 0.0021 and 0.0016, respectively) and creatinine (P= 0.0168 and 0.0018, respectively). Serum blood urea nitrogen (BUN) and creatinine are negatively associated with the abundance of ammonia-producing bacteria.

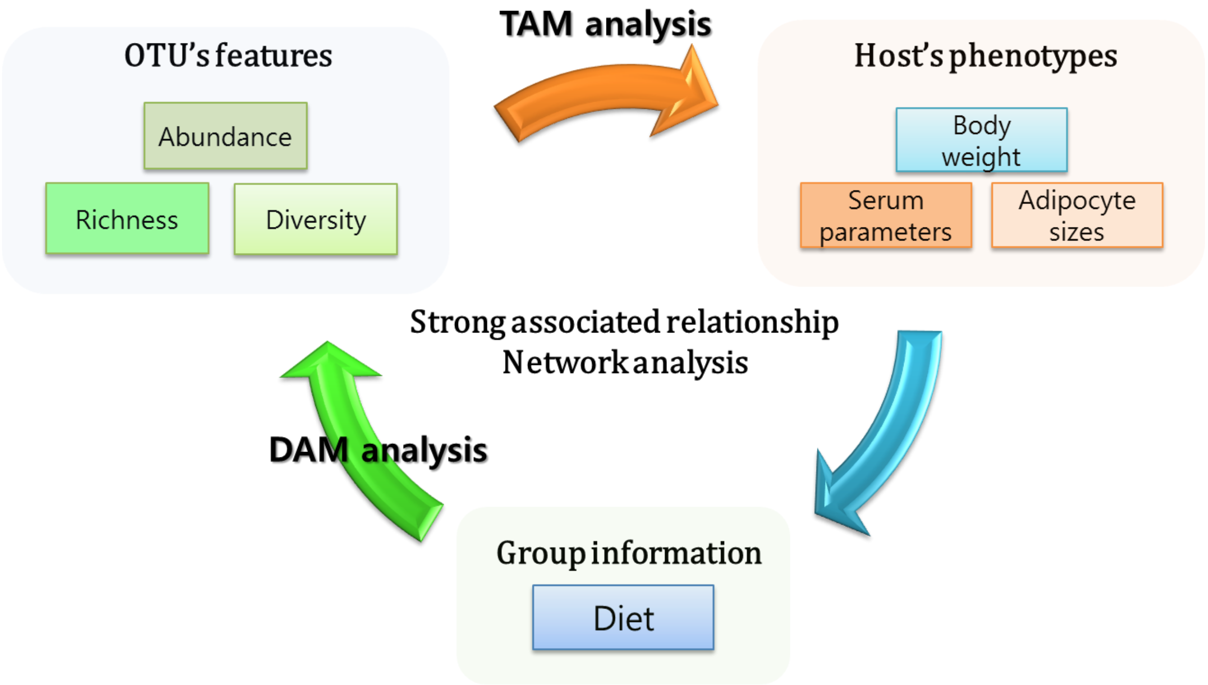

Fig. S4. Schematic diagram of DAM and TAM analysis.

Table S1. Formula of low fat diet (LFD) and high fat diet (HFD).

Product No.	D12450B (LFD)		D12492 (HFD)	
**Energy composition**	g%	Kcal%	g%	Kcal%
Protein	19.2	20.0	26	20
Carbohydrate	67.3	70.0	26	20
Fat	4.3	10.0	35	60
Total	-	100.0	-	100.0
Kcal/g	3.85	-	5.24	-
**Ingredient**	g	Kcal	g	Kcal
Casein	200	800	200	800
L-Cystine	3	12	3	12
Corn starch	315	1260	0	0
Maltodextrin 10	35	140	125	500
Sucrose	350	1400	68.8	275
Cellulose, BW200	50	0	50	0
Soybean oil	25	225	25	225
Lard	20	180	245	2205
Mineral mix S10026	10	0	10	0
Dicalcium phosphate	13	0	13	0
Calcium carbonate	5.5	0	5.5	0
Potassium citrate, 1 H2O	16.5	0	16.5	0
Vitamin mix V10001	10	40	10	40
Choline bitartrate	2	0	2	0
FD&C yellow dye #5	0.05	0	0	0
FD&C blue dye #1	0	0	0.05	0
**Total**	1055.05	4057	773.85	4057

Table S2. Dietary intervention effects on body weight in mice for 9 weeks (g).

Weeks	HFD	HFD+P	HFD+P+G	LFD
**0**	21.59±0.38	21.51±0.30	21.29±0.35	21.56±0.36
**1**	21.89±0.46	22.11±0.29	21.70±0.38	21.71±0.42
**2**	22.98±0.59	22.97±0.47	22.83±0.51	21.74±0.40*
**3**	23.95±0.72	24.39±0.50	23.75±0.65	22.35±0.43
**4**	26.27±0.72	26.04±0.66	25.03±0.62	23.51±0.45**
**5**	28.24±0.79	27.32±0.79	26.16±0.79	23.96±0.45***
**6**	29.84±0.76	28.64±0.88	27.26±1.01*	24.77±0.52***
**7**	31.98±0.72	30.22±1.01	28.46±1.24*	25.44±0.63***
**8**	33.70±0.71	32.02±1.15	29.43±1.23**	26.44±0.63***
**9**	34.73±0.65	33.06±1.28	30.46±1.21**	27.09±0.63***

Male C57BL/6J mice were fed a low fat diet (LFD), a high fat diet (HFD), a HFD with 500mg/kg BW of probiotics mixture (HFD+P) or a HFD with Probiotics mixture + 1,000mg/kg BW of *Garcinia cambogia* extract (HFD+P+G) for 9 weeks. Body weight was significantly smaller in LFD mice after 4 weeks and HFD+P+G mice after 6 weeks, respectively. Data are represented as the mean ± SEM and were analyzed using pairwise t-test (n=7 in each group). *P<0.05, **P<0.01, ***P<0.001 compared with HFD fed mice.

Table S3. Dietary intervention effects on body weight gain and fat mass accumulation.

	HFD	HFD+P	HFD+P+G	LFD
**Body weight gain (g)**	13.14±0.32	11.54±1.17	9.18±0.91**	5.53±0.33***
**Perirenal fat pad mass (%)**	2.34±0.22	1.92±0.2	2.01±0.19	1.19±0.13**
**Epididymal fat pad mass (%)**	5.97±0.14	5.57±0.71	5.77±0.53	2.98±0.23**
**Subcutaneous fat pad mass (%)**	5.76±0.29	4.62±0.67	4.58±0.31	2.36±0.21***
**Mesenteric fat pad mass (%)**	1.11±0.07	1.05±0.15	2.04±0.63	0.74±0.09
**Fat pad mass (%)**	15.18±0.33	13.15±1.64	14.41±1.09	7.27±0.63**
**Perirenal adipocyte mean size (μm^2)**	29,984 ±3,632	22,028 ±2,464*	18,963 ±1,511*	12,364 ±1,230**
**Epididymal adipocyte mean size (μm^2)**	24,139 ±1,722	18,515 ±811**	16,141 ±998***	11,691 ±564***
**Subcutaneous adipocyte mean size (μm^2)**	16,125 ±1,220	13,888 ±938	10,869 ±626***	7,757 ±516***
**Mesenteric adipocyte mean size (μm^2)**	9,886 ±526	6,991 ±747**	5,889 ±394***	4,984 ±240***

Four different adipocyte tissues were removed and weighed after 9 weeks. Body weight gain significantly reduced in LFD mice, HFD+P mice and HFD+P+G mice compared with HFD mice. All fat pad mass proportions and a subcutaneous fat pad mass proportion to body weight significantly reduced in LFD mice and HFD+P+G mice from HFD mice, respectively. Data are represented as the mean ± SEM and are analyzed using pairwise t-test (n=7). *P<0.05, **P<0.01, ***P<0.001 compared with HFD fed mice.

Table S4. Spearman correlation between C. aminophilum and other commonly identified bacteria from DAM and TAM analysis.

Commonly identified species in DAM and TAM	Spearman's correlation
Streptococcus thermophiles	0.824958
Clostridium innocuum	0.76602
Bacillus sp.	0.734613
Clostridium leptum	0.731225
Dorea formicigenerans	0.725579
Oribacterium sp. oral taxon 078	0.716544
Desulfotomaculum indicum	0.715415
Clostridium baratii	0.704122
Clostridium scindens	0.699605
anaerobic bacterium EtOH8	0.687156
Acetobacterium woodii	0.683988
Eubacterium fissicatena	0.679277
Coprococcus eutactus	0.677806
Alicyclobacillus kakegawensis	0.67429
Nitrococcus mobilis	0.670652
Clostridium propionicum	0.667984
Eubacterium sp. WAL 18692	0.647259
Anaerobranca gottschalkii	0.643139
Oxobacter pfennigii	0.638622
Clostridium cadaveris	0.628458
Clostridium lituseburense	0.623012
Clostridium histolyticum	0.622812
Ruminococcus bromii	0.619424
Anaerotruncus colihominis	0.613778
Streptococcus pleomorphus	0.613765
Ethanoligenens harbinense	0.599097
Peptoniphilus indolicus	0.594579
Candidatus Arthromitus sp. SFB-mouse	0.57651
Eubacterium rectale	0.575381
Eubacterium ruminantium	0.565217
Clostridium aminobutyricum	0.534726
Veillonella ratti	0.518916
Tannerella forsythia	0.517787
Sisymbrium irio	0.514399

rumen bacterium NK4A66	0.499718
Butyricimonas synergistica	0.499718
Lactobacillus sharpeae	0.49633
Anaerostipes caccae	0.483907
Clostridium hathewayi	0.482778
Clostridium phytofermentans	0.47939
Peptoniphilus asaccharolyticus	0.469259
Megamonas hypermegale	0.460192
Peptostreptococcus anaerobius	0.44664
Lactococcus piscium	0.432877
Parabacteroides distasonis	0.411632
Clostridium bartlettii	0.40227
Ornithobacterium rhinotracheale	0.333928

Method S1. Supplementary Methods

In this study, 16s rRNA taxonomic analysis, using a diet-induced obesity mice experiment that consists of four diet groups, was performed in order to identify causative bacterial species related to obesity. As means of detection, two types of statistical methods were utilized: 1) identifying differentially abundant microbiota (DAM); 2) identifying obesity trait associated microbiota (TAM); and 3) comparing DAM and TAM analysis results. In addition, network analysis was employed in order to consider the comprehensive information on gut microbiota features of diet-microbe, host-microbe, and microbe-microbe.

Statistical analysis for finding differentially abundant microbiota (DAM) corresponding to diet

To detect diet affected microbes, negative binomial distribution based generalized linear model was employed. Statistical methods for detecting differentially abundant biomarker are well-developed in RNA-seq field. Recent studies demonstrate applications of statistical methods in differentially abundant microbiome detection, under given conditions [1,2]. Applying these methods on our datasets is straightforward and reasonable, since both data (RNA-seq based transcriptome data and Miseq based microbiome data) composed with count-based N (Number of samples) by P (Number of biomarkers) matrix; the data structures are almost identical to that of previous works. Here, Negative bionomical based generalized linear model (GLM) can be used to detect differentially abundant microbiota (DAM), with TMM normalization. Using these relative abundances in each microbe, Analysis of Deviance (ANODEV) can be applied in order to detect differentially abundant microbiome given conditions.

$$\log(\theta_{ijk}) = \mu_j + \tau_{ij} + \beta_{jk} \qquad \text{(Eq. S1)}$$

$$\log(E(OTU\ abundance)) = \mu + Diet \qquad \text{(Eq. S2)}$$

where, i is treatment, j is gene, and k is individual. Based on the log-link function and related linear predictor (1, 2), we can test significant changes in abundance corresponding to diet. Test results were adjusted by Benjamini-Hochberg method.

Statistical analysis for detecting trait associated microbiota (TAM)

Next, simple linear regression model was used for detecting trait associated microbiota (TAM). As shown in (3), by setting the trait as response variable and abundance of microbiome as explanatory variable, we can perform association test between microbiome and obesity related traits.

$$Trait_i = \beta_0 + \beta_1 \cdot \mathrm{Abundance}_{1i} + \varepsilon_i, \varepsilon_i \sim N(0, \sigma^2) \qquad \text{(Eq. S3)}$$

Under the null hypothesis, $H_0: \beta_1 = 0$, association tests were performed on each microbiome. Finally, p-values were adjusted by Benjamini-Hochberg method in order to consider multiple testing problem.

References

1 McMurdie, P. J. & Holmes, S. Waste not, want not: why rarefying microbiome data is inadmissible. *PLoS Computational Biology* **10**, e1003531 (2014).

2 White, J. R., Nagarajan, N. & Pop, M. Statistical methods for detecting differentially abundant features in clinical metagenomic samples. *PLoS Computational Biology* **5**, e1000352 (2009).
